# HIV Infection and Bone Abnormalities

**DOI:** 10.2174/1874325001711010777

**Published:** 2017-08-21

**Authors:** Aamir N. Ahmad, Shahid N. Ahmad, Nafees Ahmad

**Affiliations:** 1 Department of Immunobiology, College of Medicine, University of Arizona, Tucson, Arizona, AZ, USA; 2 Department of Internal Medicine, University of South Dakota Sanford School of Medicine, Rapid City, South Dakota, USA

**Keywords:** HIV, Antiretroviral therapy, Bone mineral density, Osteoporosis, Osteopenia, Vitamin D, HIV Gp120, Osteoclast, Osteoblast

## Abstract

More than 36 million people are living with human immunodeficiency virus (HIV) infection worldwide and 50% of them have access to antiretroviral therapy (ART). While recent advances in HIV therapy have reduced the viral load, restored CD4 T cell counts and decreased opportunistic infections, several bone-related abnormalities such as low bone mineral density (BMD), osteoporosis, osteopenia, osteomalacia and fractures have emerged in HIV-infected individuals. Of all classes of antiretroviral agents, HIV protease inhibitors used in ART combination showed a higher frequency of osteopenia, osteoporosis and low BMD in HIV-infected patients. Although the mechanisms of HIV and/or ART associated bone abnormalities are not known, it is believed that the damage is caused by a complex interaction of T lymphocytes with osteoclasts and osteoblasts, likely influenced by both HIV and ART. In addition, infection of osteoclasts and bone marrow stromal cells by HIV, including HIV Gp120 induced apoptosis of osteoblasts and release of proinflammatory cytokines have been implicated in impairment of bone development and maturation. Several of the newer antiretroviral agents currently used in ART combination, including the widely used tenofovir in different formulations show relative adverse effects on BMD. In this context, switching the HIV-regimen from tenofovir disoproxil fumarate (TDF) to tenofovir alafenamide (TAF) showed improvement in BMD of HIV-infected patients. In addition, inclusion of integrase inhibitor in ART combination is associated with improved BMD in patients. Furthermore, supplementation of vitamin D and calcium with the initiation of ART may mitigate bone loss. Therefore, levels of vitamin D and calcium should be part of the evaluation of HIV-infected patients.

## 1 INTRODUCTION

There are about 36.7 million people living with human immunodeficiency virus (HIV) infection worldwide [1]. While 2.1 million people were newly infected with HIV-1 in 2015, which has not changed among adults since 2010, 1.1 million people have died of HIV/AIDS in 2015, showing a decline of 45% since 2005 worldwide [1]. Since the start of the epidemic until the end of 2015, 78 million people have been infected with HIV and 35 million people have died of HIV/AIDS worldwide [1]. More importantly, 18.2 million HIV-infected individuals have access to antiretroviral therapy (ART) by June 2016, which is almost 50% of the HIV-infected population worldwide [1]. In the United States, the Center for Disease Control and Prevention (CDC) estimates that approximately 1.2 million people are living with HIV, and males accounted for 76% of the HIV-infected population, and more than one-half million people have died with HIV/AIDS. New HIV infections in the recent years in the United States have remained relatively stable at around 50,000 per year, whereas HIV-related death rates have declined significantly. Recent advances in HIV therapy (antiretroviral therapy, ART) have reduced the viral load, increased CD4 T cell counts and slowed progression of the HIV disease in many HIV infected-patients and appears to be responsible for a dramatic improvement of these patients lives. However, toxicity and the development of resistance remain the concerns. In addition to side effects of ART, several complications of ART include lipoatrophy, hypercholesterolemia, low HDL, hypertriglyceridemia, insulin resistance, impaired glucose tolerance, cardiovascular disease, lactic acidosis, and bone abnormalities.

Long term infection with HIV and use of ART in HIV-infected patients are associated with several bone related abnormalities such as low bone mineral density (BMD), osteomalacia, osteopenia, osteoporosis, osteonecrosis, fracture and other bone disorders [2-5]. While bone disorders are multifactorial in nature, nutritional deficiencies such as vitamin D levels and other classical risk factors for bone disorders, including smoking and tobacco use, which are prevalent in HIV-infected individuals, may exacerbate bone related abnormalities in these patients who are on long term ART [6]. A better understanding of the etiology and pathogenesis of bone related disorders in HIV-infected people who are now living longer because of ART may provide useful information that could be included in the treatment strategies of these aging HIV-infected individuals. This article will review the bone conditions and abnormalities associated with HIV infection and use of ART in HIV-infected patients.

## 2 HIV INFECTION AND BONE MINERAL DENSITY

Several studies have reported low bone mineral density (BMD) with increased risk of osteoporosis, osteopenia and, osteomalacia in HIV-infected individuals, including men, women, younger and older patients and vertically infected children [7-16]. A recent study evaluating 58 HIV-infected children and adolescents between the ages of 5.3 to 18.3 years, including 63.3% girls found an increased risk for lower BMD and lower levels of vitamin D serum concentration [17] and the loss in BMD is associated with the levels of vitamin D binding protein [18]. These and other studies [19] suggest that vitamin D levels should be included in the overall evaluation of HIV-infected patients. In addition, HIV infection is associated with poor bone material properties, independent of BMD [20] and low BMD is associated with an increased risk of fracture [21]. The prevalence of osteopenia and osteoporosis independent of ART was evaluated in HIV-infected male patients and found that ART is not a predictor for the risk of osteopenia and osteoporosis in HIV-infected patients [22], suggesting that HIV infection is associated with these bone abnormalities. These studies show an association of HIV infection with bone abnormalities, including low BMD, osteopenia, osteomalacia and osteoporosis in infected patients [23]. However, the mechanisms of HIV induced bone abnormalities are not well understood. We will explore the literature to determine the potential mechanisms of HIV involvement in bone disorders in HIV-infected patients.

While the mechanisms of bone loss or abnormalities during HIV infection are not known, it is hypothesized that the damage may be due to a complex interaction of the T cells with osteoclasts and osteoblasts likely influenced by both HIV and ART [16]. One potential and important mechanism of HIV induced bone disorders could be due to HIV infection of osteoclasts that are derived from monocytes and are resident macrophages in the bone tissue [24]. Since osteoclasts are required for maintenance, repair and remodeling of bones, infection of osteoclasts by HIV leads to its differentiation [24] and most likely contributes to osteolytic disease in HIV-infected patients. Several additional studies have been performed to determine the role of HIV proteins on bone disorders. HIV Gp120, the envelope protein of the virus that binds to CD4 receptor and CCR5 or CXCR5 coreceptors, was shown to induce apoptosis of osteoblasts [25, 26], including a significant upregulation of proinflammatory cytokine, TNF-α, and Wnt/β-catenin signaling [26] likely contributing to bone loss. In addition, HIV Gag P-55, the precursor protein for HIV matrix, capsid and nucleocapsid proteins, was also found to decrease the level of osteogenesis in mesenchymal stem cells, seemingly playing a role in reducing BMD [27]. These studies suggest that HIV and its gene products such as envelope Gp120 and Gag p55 play important roles in interfering with bone development and maturation. Furthermore, HIV-infected patients who receive ART, which may further complicate bone related abnormalities. A recent study that included HIV-infected postmenopausal women receiving ART which included the HIV protease inhibitor, ritonavir, showed higher bone turnover markers and increased differentiation of osteoclast-like cells from adherent peripheral blood mononuclear cells with increased risk of bone loss [28]. Another study showed that an HIV protease inhibitor increased the rate of apoptosis and impairment of osteogenic markers in an osteoblast like cell line [29]. These studies suggest that ART may play a role in bone related disorders in HIV-infected individuals, which will be discussed later in this article.

One important investigation could be to determine if HIV infection of bone marrow cells may contribute to bone related disorders. In this context, HIV infection of bone marrow stromal cells has been demonstrated in several studies [30-32]. In HIV-infected patients, the bone marrow CD34+ progenitor cells were found to have an impaired T cell differentiation due to the production of proinflammatory cytokines [33]. In addition, B cells in HIV-infected patients express higher levels of the receptor activator of NFκ−B ligand (RANKL) and lower levels of osteoprotegerin (OPG) that influence osteoclastic bone resorption [34]. In a recent study evaluating the negative effect on bone acquisition caused by HIV infection early in life, it was found that T cell activation was associated with decreased number of osteogenic precursors and lower bone mass and strength [35]. Taken together, these studies suggest that HIV infection of bone marrow cells may have a role in bone development and reducing bone mineral density, contributing to many abnormalities associated with the bones.

## 3 ANTIRETROVIRAL THERAPY AND BONE MINERAL DENSITY

HIV-infected patients are placed on ART as soon as their HIV status is known. However, long term use of ART has been shown to be associated with many disorders, including lipoatrophy, hypercholesterolemia, low HDL, hypertriglyceridemia, insulin resistance, impaired glucose tolerance, cardiovascular disease, lactic acidosis and bone disorders. Several studies have also shown that the frequency of osteoporosis was higher in HIV-infected individuals receiving ART compared with uninfected individuals [36], including a decrease in BMD [37-43]. Since HIV-infected patients are on ART indefinitely, continuous administration of ART has been shown to be associated with decreased BMD and an increased risk of fractures compared with intermittent CD4 T cell count guided ART [44]. Earlier studies evaluated the effects of ART that included two nucleoside reverse transcriptase inhibitors (NRTI) and one non-nucleoside reverse transcriptase inhibitor (NNRTI) or a protease inhibitor (PI) and found that ART influenced bone turnover [45] and reduction in BMD [46-48]. In addition, patients receiving ART that included a protease inhibitor showed a higher incidence of osteoporosis than those without a protease inhibitor [49]. This finding was further supported by several studies showing that 50% of HIV-infected patients receiving PI inhibitor had osteopenia, 21% had osteoporosis [10] and 71% of patients had decreased BMD [49-51]. In HIV-infected women, HIV infection was associated with lower BMD independent of other known risk factors for decreased BMD [52]. In addition, protease inhibitor-containing ART and particularly longer use of lopinavir were associated with lower BMD, whereas use of efavirenz was associated with higher BMD [52].

There are more than thirty antiretroviral agents from different classes approved by FDA available for use in combination for HIV regimen in patients. Several studies have evaluated the relative effects of these antiretroviral agents on bone abnormalities in infected patients. In a study performed on HIV-infected patients in Korea, osteoporosis was associated with both abacavir- and zidovudine-based HIV regimen, however, zidovudine associated osteoporosis was seen mainly after 1 year of treatment, whereas abacavir had adverse osteological effects in less than 1 year [52]. Recent studies addressing the BMD issues in HIV-infected women from Sub-Saharan Africa found that HIV- infected women on ART had a 2-3% decrease in their BMD [53]. Contrary to this, some studies have found that there was no reduction or difference in BMD in those HIV-infected patients who were treated with ART [54, 55].

A meta-analysis of 2,210 patients suggested that changing the HIV-regimen to tenofovir disoproxil fumarate (TDF) is associated with reduction in BMD [56] and switching from TDF to tenofovir alafenamide (TAF) led to improved BMD [57, 58]. A recent two double blind phase 3 trial of 1,733 ART naïve HIV-infected individuals found that a TAF based regimen had better virologic efficacy and less impact on BMD compared with a TDF based regimen formulated with similar antiretroviral agents [59]. In a comparative analysis of HIV-infected antiretroviral treatment-naïve African American patients, it was found that the ART combination of efavirenz, emtricitabine, and TDF was associated with reduction in BMD and maintained the levels of vitamin D compared with the ART combination containing PI, raltegravir, darunavir, and ritonavir [60]. In addition, vitamin D levels are also associated with lower BMD in those who receive efavirenz or lopinavir/ritonavir [61]. In another comparative analysis of specific ART, it was found that TDF/emtricitabine plus atazanavir/ritonavir, darunavir/ritonavir (DRV/r), or raltegravir were associated with bone loss, but the lowest was with raltegravir [62]. Furthermore, supplementation with vitamin D and calcium with the initiation of ART efavirenz/emtricitabine/TDF may mitigate bone loss in HIV-infected patients [63]. In AIDS Clinical Trials Group A5303 study, it was found that maravioroc (CCR5 inhibitor) containing ART was associated with less bone loss at hip and lumbar spine than tenofovir disoproxil fumarate containing ART, suggesting that maravioroc may be an option in ART to reduce bone loss [64].

Now the question is that how should we follow HIV-infected patients on ART with respect to their loss of BMD. In general, dual-energy X-ray absorptiometry (DEXA) is used to assess BMD. Some studies suggest that infected patients should be followed for serum bone specific alkaline phosphatase and urinary N-terminal telopeptide [65] along with levels of osteoprotegerin [66], which may be predictive markers for loss of BMD and development of osteoporosis. Some studies recommend that instead of using DEXA, quantitative ultrasound (QUS) can be used to assess BMD initially in HIV-infected patients to avoid unnecessary radiation exposure from DEXA because many patients may not benefit from it [67, 68].

## CONCLUSION

Recent advances in HIV therapy have improved the quality and longevity of HIV-infected patients’ lives due to suppression of viral load, improvement of CD4 T cell counts and reduction to almost elimination of opportunistic infection in developed countries, especially United States. Moreover, more than 50% of HIV infected people worldwide now have access to ART. However, HIV-infected patients receiving ART are experiencing many complications, including bone disorders. HIV infected people experience low BMD and are at increased risk for osteopenia, osteoporosis, osteomalacia and fractures. Furthermore, use of antiretroviral agents further exacerbate these bone abnormalities. Current recommendation for ART combination includes at least three drugs from two different classes of antiretroviral agents; nucleoside reverse transcriptase inhibitors, non-nucleoside reverse transcriptase inhibitors, integrase inhibitors and protease inhibitors. Of all these classes of antiretroviral agents, protease inhibitors showed a higher frequency of osteoporosis, osteopenia and low BMD. Several of the newer ART agents are also associated with low BMD and other bone abnormalities. However, switching the HIV-regimen from tenofovir disoproxil fumarate (TDF) to Tenofovir alafenamide (TAF) showed improvement in BMD of HIV-infected people. Also, use of integrase inhibitor in ART combination improved BMD in patients. It was found useful to supplement vitamin D and calcium with ART to reduce bone loss in HIV-infected patients.
